# Editorial: New trends in type 2 diabetes diagnosis and management in primary care, volume II

**DOI:** 10.3389/fmed.2025.1771093

**Published:** 2026-01-12

**Authors:** Aleksandra Klisic, I-Shiang Tzeng, Filiz Mercantepe

**Affiliations:** 1 Faculty of Medicine, University of Montenegro, Podgorica, Montenegro; 2 Center for Laboratory Diagnostics, Primary Health Care Center, Podgorica, Montenegro; 3 Department of Statistics, School of Business, National Taipei University, New Taipei, Taiwan; 4 Department of Endocrinology and Metabolism, Faculty of Medicine, Recep Tayyip Erdogan University, Rize, Turkey

**Keywords:** cardiovascular risk, insulin resistance, primary care, screening, type 2 diabetes

Type 2 diabetes mellitus (T2DM) continues to represent one of the most substantial and persistent global health challenges, with its burden extending far beyond glycemic control to encompass cardiovascular disease, microvascular complications, healthcare utilization, and socioeconomic consequences (1). As the prevalence of T2DM rises worldwide, primary care settings remain the cornerstone of early detection, risk stratification, long-term management, and patient-centered prevention strategies (2). In this context, primary care professionals are increasingly required to integrate evolving diagnostic tools, therapeutic innovations, and multidisciplinary approaches into everyday clinical practice.

Following the strong interest generated by the first volume of *New Trends in Type 2 Diabetes Diagnosis and Management in Primary Care*, the present Volume II was conceived to capture the rapid developments that have emerged in recent years. Since the publication of the initial Research Topic, the field has undergone significant transformation, driven by advances in predictive modeling, growing attention to real-world healthcare delivery, digital and artificial intelligence–assisted tools, and a deeper understanding of behavioral, psychosocial, and system-level determinants of diabetes outcomes. Together, these changes underscore the need for updated, practice-oriented insights tailored specifically to the realities of primary care.

**Figure 1:**
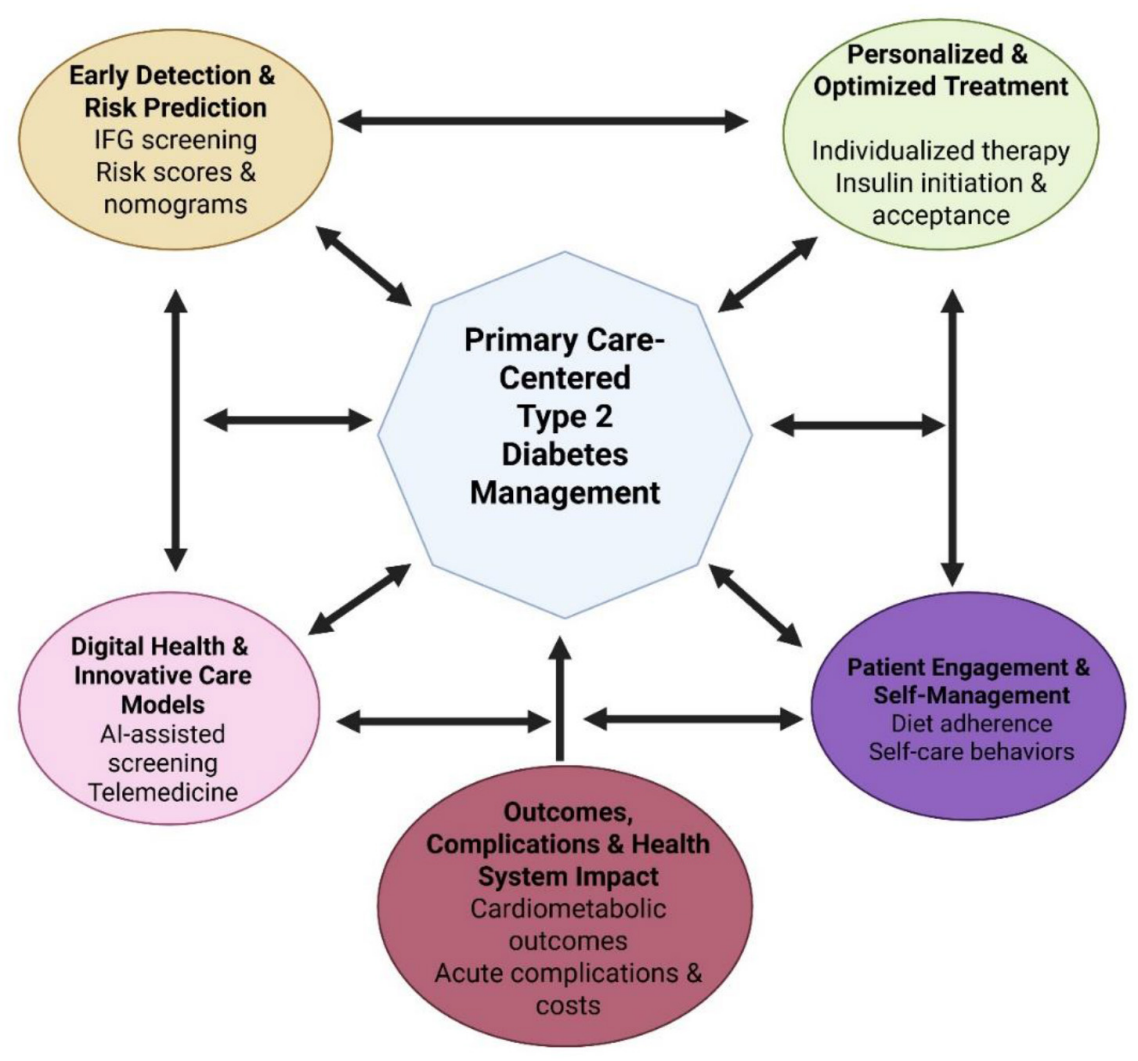
A primary care-centered framework for contemporary diagnosis and management of type 2 diabetes.

The articles included in this Research Topic collectively address several interconnected dimensions of contemporary diabetes care. The scope and thematic structure of this Research Topic are summarized in Figure 1, which illustrates a primary care–centered framework for contemporary T2DM management. A central theme across multiple contributions is the importance of early identification of dysglycemia and risk stratification. Predictive approaches that combine readily available clinical and biochemical variables demonstrate the potential to identify individuals at high risk for impaired fasting glucose and future diabetes, offering pragmatic tools that can be implemented in resource-constrained primary care environments “*A study on predicting impaired fasting glucose risk in Chinese adults based on individual characteristics*” (Lin et al.). Such strategies align closely with the shift toward prevention-focused care and personalized risk communication.

Another prominent focus of this volume relates to healthcare utilization, system-level outcomes, and economic implications of diabetes management. Analyses of inpatient expenditure and patterns of care provide valuable insight into the drivers of healthcare costs, highlighting the influence of comorbidities, disease complexity, and care pathways “*In-patient expenditure between 2011 and 2021 for patients with type 2 diabetes mellitus: a hospital-based multicenter retrospective study in southwest China*” (He et al.). In parallel, the examination of healthcare delivery during the COVID-19 pandemic offers a unique natural experiment, illustrating how reductions in structured chronic disease management programs may lead to compensatory increases in general practitioner visits and hospital-based care “*Downsizing chronic disease management programs for type 2 diabetes patients during the COVID-19 pandemic: changes in healthcare utilization patterns*” (Rijpkema et al.). These findings reinforce the critical role of sustained, well-organized primary care programs in mitigating downstream healthcare burden.

Several contributions extend beyond biomedical outcomes to explore the human and behavioral dimensions of diabetes management, which are particularly salient in primary care. Studies addressing dietary adherence, self-care practices, and socioeconomic determinants reveal persistently high rates of non-adherence and identify structural barriers such as food insecurity, limited health literacy, and restricted access to healthcare services “*Adherence to recommended diet among patients with diabetes mellitus type 2 on follow-up at Adama Hospital Medical College, Ethiopia*” (Abose et al.); “*Adherence to diabetic self-care management and associated factors among type 2 diabetic patients in North Shewa Zone public hospitals in Amhara Region, Ethiopia*” (Endale et al.). Importantly, these findings emphasize that effective diabetes management cannot be achieved through pharmacological strategies alone, but requires integrated approaches combining education, social support, and health system responsiveness. In this context, qualitative research focusing on interprofessional colla borative care highlights the practical challenges faced by newly trained healthcare professionals when translating interprofessional education into real-world diabetes management within primary care settings “*A grounded theory research protocol on an attempt to practice interprofessional collaborative care by a primary care clinic health professional fresh graduate in diabetes care*” (Num et al.).

Innovation in care delivery is another defining feature of this Research Topic. Qualitative investigations into artificial intelligence–assisted diabetic retinopathy screening offer valuable real-world perspectives from general practice staff, shedding light on feasibility, trust, and implementation challenges “*Valuable insights into general practice staff's experiences and perspectives on AI-assisted diabetic retinopathy screening—An interview study*” (Krogh et al.). These insights are essential as digital health tools increasingly transition from pilot projects to routine clinical use. Similarly, emerging approaches to insulin delivery, such as needle-free injection systems, address psychological insulin resistance and patient acceptance—factors that often determine the success or failure of intensification strategies in everyday practice “*Effect of needle-free injection on psychological insulin resistance and insulin dosage in patients with type 2 diabetes*” (Wang et al.).

The scope of this volume also encompasses acute and high-risk clinical scenarios that intersect with primary care decision-making. Studies examining prognostic biomarkers in diabetic ketoacidosis “*Association between blood urea nitrogen to serum albumin ratio and in-hospital mortality in critical patients with diabetic ketoacidosis*” (Chen et al.), longitudinal changes in resting heart rate as predictors of adverse outcomes “*Effects of temporal changes in resting heart rate on future diabetes-related outcomes*” (Gao et al.), and complex endocrine emergencies “*Case report: management of a young male patient with diabetic ketoacidosis and thyroid storm*” (Huang et al.) highlight the need for vigilance, timely referral, and interdisciplinary collaboration. Although such conditions may present in hospital settings, early recognition and appropriate triage frequently depend on primary care awareness and continuity of care.

Taken together, the contributions in this Research Topic underscore a central message: modern diabetes care in primary care settings is no longer defined solely by glycemic targets, but by a broader, more nuanced framework that integrates prediction, prevention, patient experience, system efficiency, and long-term outcomes. The diversity of methodologies represented—ranging from large database analyses and cohort studies to qualitative research and clinical trials—reflects the multifaceted nature of diabetes management and the necessity of addressing it from multiple angles.

As Topic Editor, we believe that this Research Topic provides timely and clinically relevant insights for primary care physicians, researchers, and policymakers alike. By bridging evidence-based innovations with real-world practice, *New trends in type 2 diabetes diagnosis and management in primary care, volume II* aims to support more effective, equitable, and patient-centered diabetes care. We hope that the perspectives presented here will stimulate further research, encourage interdisciplinary collaboration, and ultimately contribute to improved outcomes for individuals living with type 2 diabetes.

Figure 1 illustrates a conceptual, primary care-centered model for contemporary type 2 diabetes management. Primary care is positioned at the core of diabetes care, integrating early detection and risk prediction, personalized and optimized treatment strategies, patient engagement and self-management, digital health innovations, and the monitoring of outcomes, complications, and healthcare system impact. The interconnected domains reflect the multifaceted approaches addressed in this Research Topic and highlight the central role of primary care in coordinating prevention, individualized care, real-world implementation, and long-term outcome optimization for individuals with type 2 diabetes.
